# Comments on “Determination of Mercury, Cadmium, Lead, Zinc, Selenium and Iron by ICP-OES in Mushroom Samples from Around Thermal Power Plant in Muğla, Turkey”. doi:10.1007/s00128-011-0357-1

**DOI:** 10.1007/s00128-012-0566-2

**Published:** 2012-02-28

**Authors:** Jerzy Falandysz

**Affiliations:** Research Group of Environmental Chemistry, Ecotoxicology and Food Toxicology, Institute of Environmental Sciences and Public Health, University of Gdańsk, 18 Sobieskiego Str., 80-952 Gdańsk, Poland

**Keywords:** Fungi, Mushrooms, ICP-OES, Selenium, Trace metals, Wild foods

## Abstract

In several articles on trace elements in mushrooms erroneous data were published on minerals sequestered in fruiting bodies. The biased analytical data published gave a false picture on the composition and nutritional value of mushrooms with respect to minerals. Wild mushrooms are relatively rich in trace elements and some species can hyperaccumulate certain metals. Selenium as reported in the referenced article is discussed in light of typical Se concentrations determined using validated methods as reported by other authors.

I read the article on contents of mercury, cadmium, lead, zinc, selenium and iron in mushrooms from an area affected by a thermal power plant fueled with hard coal. I came to this report with a particular interest with respect to Se concentrations. Selenium is vital to human health and a good bio-available source of this element from foods is needed to maintain good health (Jarzyńska and Falandysz 2011a). Selenium is found in different chemical species, has a relatively narrow margin of safety between an adequate, inadequate and excess intake, is highly potent and has great health impact. Hence, accurate data on Se content of foods are essential both in research and applicable surveys to enable health risk assessments to be carried out.

Both cultivated and wild mushrooms can be important constituents of the market basket, especially to vegans and vegetarians who may otherwise lack certain nutrients from their diet. Consumption rates of mushrooms vary largely between the nations, ethnic groups and regions of the world (Zhang et al. 2010). Many mushrooms are rich in K, P, Rb, Cu, Mn and Zn and in addition some species are abundant in a specific element or elements e.g. Ag, Cd, Cu, Fe, Hg, Se, V (Borovička and Řanda 2007; Borovička et al. 2010; Costa-Silva et al. 2011; Falandysz et al. 2001, 2002, 2003, 2004, 2007a, b, c, d, 2008a, b, 2012; Gucia et al. 2012; Jarzyńska et al. 2011). All mushrooms contain Se in flesh but a few edible wild mushrooms are specifically rich in Se (>10 μg/g dry weight), while in many the content is <1.0 μg/g dw (Fig. 1; Falandysz 2008).

**Fig. 1:**
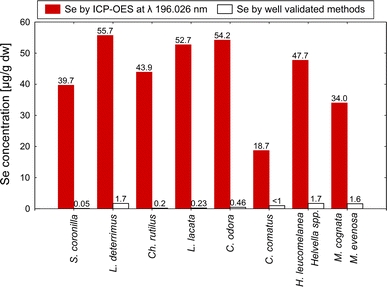
The values of Se concentrations reported in certain mushrooms as determined using ICP-OES by Kula et al. (2011) (*left bar*) and by other researchers when using generally accepted methods (*right bar*; as cited after Falandysz (2008) and Mandić et al. (1991); the mushroom names are such as *Stropharia coronilla* (*S*. *coronilla*); *Lactarius deterrimus* (*L*. *deterrimus*); *Chroogompus rutilus* (*Ch*. *rutilus*); *Laccaria lacata* (*L. laccata*); *Clitocybe odora var. alba* (*C*. *odora*); *Coprinus comatus* (*C*. *comatus*); *Helvella leucomelanea* (*H*. *leucomelanea*) (*filled bar*) and various *Helvella spp*. (*empty bar*); *Melanoleuca cognata* (*M*. *cognata*) (*filled bar*) and *M. evenosa* (*empty bar*) (color figure available online)

The cultivated mushrooms (*Agaricus bisporus*), Oyster Mushrooms (*Pleurotus ostreatus*) and Shitake (*Lentinus edeodes*) are usually relatively poor in Se (Falandysz 2008). In the USA, the cultivated species: *A*. *bisporus* (the White Button Mushroom, Portabella and Baby Bella varieties), *L*. *edeodes*, *P*. *ostreatus*, *P*. *erungii* (King Trumpet), *Hipsizygus tessulatus* (Brown Beech or Brown Clamshell Mushroom), *Flammulina veluipes* (Enoki) and *Grifola* *fromulosa* (Maitake) contained Se in concentrations varying between 0.01 and 2.7 μg/g dw, and in a consignment of *A. bisporus* concentrations were 2.7 μg/g dw and in *P*. *ostreatus* 0.2 μg/g dw (Hong et al. 2011).

Some common cultivated mushrooms if developed in substratum fortified with added Se or substratum made of biomass collected from a selenoferous area or if prepared from Se-laden plants, can be enriched in Se (Bhatia et al. 2011; Falandysz 2008; Hong et al. 2011; Rodriquez-Estrada et al. 2009).

Kula et al. (2011) reported high concentrations of Se between 18.7 and 67.10 μg/g dw for all fifteen species of mushrooms examined. For some of these mushrooms, i.e. *Stropharia coronilla*, *Lactarius deterrimus*, *Chroogompus rutilus*, *Laccaria lacata*, *Clitocybe odora var. alba*, *Coprinus comatus*, *Helvella leucomelanea*, *Melanoleuca cognate*, data on Se have been reported by other authors – see review by Falandysz (2008).

Kula et al. (2011) determined Se by inductively coupled plasma – optical emissions spectroscopy (ICP-OES; ICP-AES). Their data for eight mushrooms and “reference data” for the same species determined by other authors are given in Fig. 1. These “reference values” (Fig. 1) are valid and were obtained by widely accepted analytical methods such as hydride generation – atomic absorption spectroscopy (HG AAS), instrumental neutron activation analysis (INAA) and others. The discrepancies between erroneous Se data by ICP-OES and valid by other methods are evident (Fig. 1).

Good data on Se determined by graphite furnace AAS in mushrooms such as *Panellus stipticus*, *Tricholoma terreum*, *T. virgatum*, *Entoloma sinuatum*, *Boletus edulis*, *B. luridus*, *Suillus granulatus*, *Amanita muscaria*, *A. pantherina*, *Agaricus arvensis*, *A*. *porphyrizon*, *A*. *silvicola*, *Leucoagaricus leucothites*, *L. nympharum*, *Macrolepiota procera* and *Russula foetens* from Turkey have been reported by Tuzen et al. (2007). They imply on typical concentrations of Se in soils in Turkey and fit well with Se data for the same mushrooms but collected elsewhere in the world – as examined by several researchers, and reviewed by Falandysz (2008).

The matrix effects from biological samples due to presence of carbon but also sulfur, phosphorus and bromine can cause non-spectral interferences and it can lead to high Se concentrations as determined by ICP-AES – as discussed by several authors earlier (Grindla et al. 2007; Machat et al. 2002). Determination of Se as well as of Hg by ICP-AES even after efficient digestion/oxidation of biological material (mushrooms and plant) in closed PTFE vessels using concentrated nitric acid and with aid of microwave energy can lead to imprecise and frequently inaccurate results, when compared, respectively to HG-AAS and cold vapour – atomic absorption spectroscopy (CV-AAS) (Jarzyńska et al. 2012; Jarzyńska and Falandysz 2011b).

In the analytical quality control guidelines for the publication of analytical results of chemical analysis in foodstuffs by Jorhem et al. (2011) is stated that “suspicious of poor analytical quality of published elemental results have been mounting over the years”. To help the researchers, authors and reviewers, the check-list for the description of quality control criteria was created by these authors and the QC-guidelines are given for an open distribution (Jorhem et al. 2011). In the “Analytical parameter” part of these QC-guidelines in the sampling, methodology and certified reference materials sections, the primarily points are: (i) sampling representativity, appropriate choice of method and relevance of the certified material matrix, respectively (Jorhem et al. 2011).

In reports on minerals in mushrooms information on the number of samples (single or pooled, and number of fruiting bodies – mushrooms, if pooled sample were examined) collected and examined is often lacking detail or no such information at all is provided. It is left for the reader to guess that only a single specimen (fruiting body) was collected and examined and a value of standard deviation given refers to a single result and reflects only a variability of instrumental measurements of the same sample. Representative sample collection is important and should be made a priority, and to evaluate natural variability, the sample (specimen) number has to be no fewer than 15 in order to be satisfactory (Eckschlager 1974).

Borovička and Řanda (2007) discussed recently erroneously reported concentrations of iron (Fe) in mushrooms by some authors and the remarks there seem to apply also to data reported by Kula et al. (2011).
